# A Comprehensive Survey of Research Trends in mmWave Technologies for Medical Applications

**DOI:** 10.3390/s25123706

**Published:** 2025-06-13

**Authors:** Xiaoyu Zhang, Chuhui Liu, Yanda Cheng, Zhengxiong Li, Chenhan Xu, Chuqin Huang, Ye Zhan, Wei Bo, Jun Xia, Wenyao Xu

**Affiliations:** 1 Department of Computer Science and Engineering, State University of New York at Buffalo, Amherst, NY 14068, USA; zhang376@buffalo.edu (X.Z.); chuhuili@buffalo.edu (C.L.); weibo@buffalo.edu (W.B.); 2 Department of Biomedical Engineering, State University of New York at Buffalo, Amherst, NY 14068, USA; yandache@buffalo.edu (Y.C.); chuqinhu@buffalo.edu (C.H.); junxia@buffalo.edu (J.X.); 3 Department of Computer Science and Engineering, University of Colorado Denver, Denver, CO 80204, USA; zhengxiong.li@ucdenver.edu; 4 Department of Computer Science, North Carolina State University, Raleigh, NC 27695, USA; cxu34@ncsu.edu; 5 Linde Inc., Tonawanda, NY 14150, USA; ye.zhan@linde.com

**Keywords:** mmWave technology, medical application

## Abstract

Millimeter-wave (mmWave) sensing has emerged as a promising technology for non-contact health monitoring, offering high spatial resolution, material sensitivity, and integration potential with wireless platforms. While prior work has focused on specific applications or signal processing methods, a unified understanding of how mmWave signals map to clinically relevant biomarkers remains lacking. This survey presents a full-stack review of mmWave-based medical sensing systems, encompassing signal acquisition, physical feature extraction, modeling strategies, and potential medical and healthcare uses. We introduce a taxonomy that decouples low-level mmWave signal features—such as motion, material property, and structure—from high-level biomedical biomarkers, including respiration pattern, heart rate, tissue hydration, and gait. We then classify and contrast the modeling approaches—ranging from physics-driven analytical models to machine learning techniques—that enable this mapping. Furthermore, we analyze representative studies across vital signs monitoring, cardiovascular assessment, wound evaluation, and neuro-motor disorders. By bridging wireless sensing and medical interpretation, this work offers a structured reference for designing next-generation mmWave health monitoring systems. We conclude by discussing open challenges, including model interpretability, clinical validation, and multimodal integration.

## 1. Introduction

Millimeter-wave (mmWave) technology has emerged as a transformative solution for addressing modern healthcare challenges. mmWave-based medical applications span a wide range of fields, including diagnostics, treatment, and monitoring. These technologies have garnered attention for their potential to improve patient outcomes while offering numerous advantages. (1) Non-Contact Measurements: mmWave sensor systems have been utilized to monitor vital signs such as sleep patterns [1,2,3,4] without the need for wearable sensors, enhancing user comfort and ease of use. (2) High Precision and Sensitivity: mmWave sensor systems have shown promise in providing high-resolution sensing for detecting conditions such as skin cancer [5,6,7] and wounds [8], offering a detailed view that enhances diagnostic accuracy. (3) Miniaturization and Portability: mmWave sensors are miniature and easy to integrate into portable devices for real-time and off-the-go measurements, such as monitoring blood pressure [9,10,11,12] and body temperature [13,14]. (4) Cost-Effectiveness: The low-cost nature of mmWave sensors makes them an attractive option for at-home healthcare solutions, such as blood glucose monitoring systems that empower patients to manage their health affordably [15,16,17].

The use of mmWave sensors in medical applications typically follows a multi-stage process. First, the mmWave signal interacts with the target, capturing electromagnetic properties such as phase shifts, amplitude variations, and frequency changes. Second, common mechanisms such as Frequency-Modulated Continuous Wave (FMCW) and Synthetic Aperture Radar (SAR) are employed to acquire high-resolution information. Third, physical parameters—such as structure, motion, or material properties—are extracted via signal processing techniques. Fourth, these physical parameters are further mapped to biomedical biomarkers (e.g., heart rate variability (HRV), skin moisture) using theoretical models or data-driven algorithms. Finally, the derived biomarkers are applied in medical diagnostics or monitoring.

Despite rapid progress, a critical gap remains in understanding how mmWave signal features relate to medical biomarkers. Existing surveys [18,19,20] primarily focus on the sensing hardware, signal processing methods, or general applications. However, they often overlook the fundamental distinction between mmWave physical parameters—which describe signal behavior—and biomedical biomarkers—which represent physiological or pathological conditions. More importantly, the methods used to link these two types of features remain fragmented across the literature.

**Figure 1:**
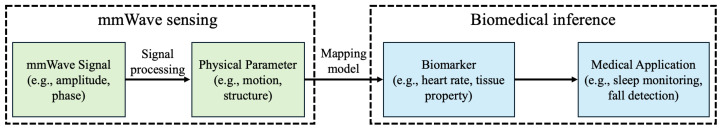
Overview of the mmWave medical sensing pipeline.

Figure 1 illustrates the full-stack pipeline of mmWave-based medical systems, from raw signal acquisition to medical application. Unlike prior reviews that focus on either hardware components or specific medical tasks, this survey provides a unified framework that bridges physical signal parameters (e.g., motion, dielectric constant) and medical biomarkers (e.g., respiration rate, heart rate, tissue hydration). We systematically review the mapping strategies—including theoretical models and machine learning approaches—that enable this transformation.

To the best of our knowledge, this is the first survey that (i) categorizes mmWave sensing features based on their physical signal origin, and (ii) links them to medical biomarkers and medical use cases through both analytical and data-driven models. By connecting low-level signal physics with high-level medical interpretation, this work serves as a reusable foundation for the design, evaluation, and translation of mmWave-enabled healthcare technologies.

## 2. Paper Search Strategy

To construct a focused and high-quality survey of mmWave-based medical applications, we adopted a multi-stage literature screening strategy. First, we performed a broad keyword search on Google Scholar using the query “mmWave for medical application”, which yielded a total of 17,900 articles published between 2010 and 2025. Second, to ensure publication quality, we filtered the results by retaining only papers published in journals with an impact factor (IF) ≥ 1.0 and conferences with a Google Scholar h5-index ≥ 30, resulting in a refined set of 2200 high-quality papers.

Next, we screened these 2200 papers based on content relevance by examining their titles and abstracts. We retained papers that explicitly mentioned application-specific keywords such as “mmWave”, “human body”, “health”, “vital sign”, “biomarker”, “wound”, “tissue”, “diabetes”, or “rehabilitation”, indicating a focus on human physiological or pathological sensing. This step resulted in a set of 523 articles with clear relevance to mmWave-based biomedical sensing.

Finally, from this pool, we manually selected 66 representative papers for in-depth analysis in the survey. Given that the primary focus of this work is to review and analyze complete mmWave-based medical application systems, we prioritized studies that demonstrated comprehensive end-to-end system designs with clearly defined technical components. The manual selection was guided by two key criteria: (1) Completeness of technical implementation: the study must present a full sensing system comprising all three essential modules—a hardware configuration module, a signal processing module, and a medical functionality module that connects the extracted mmWave features to clinically relevant biomarkers or outcomes; (2) Experimental validation: the system must be supported by a well-defined evaluation process involving human subjects, tissue phantoms, or clinically relevant samples, demonstrating the feasibility, robustness, and potential applicability of the proposed solution in real-world medical contexts.

By applying these criteria, the 66 selected papers reflect a representative subset of the field, covering diverse biomarker types, clinical objectives, and sensing strategies. These works, together, form a comprehensive foundation for understanding how mmWave systems are developed and deployed for real-world biomedical applications.

## 3. Overview of mmWave-Based Medical System

**Figure 2:**
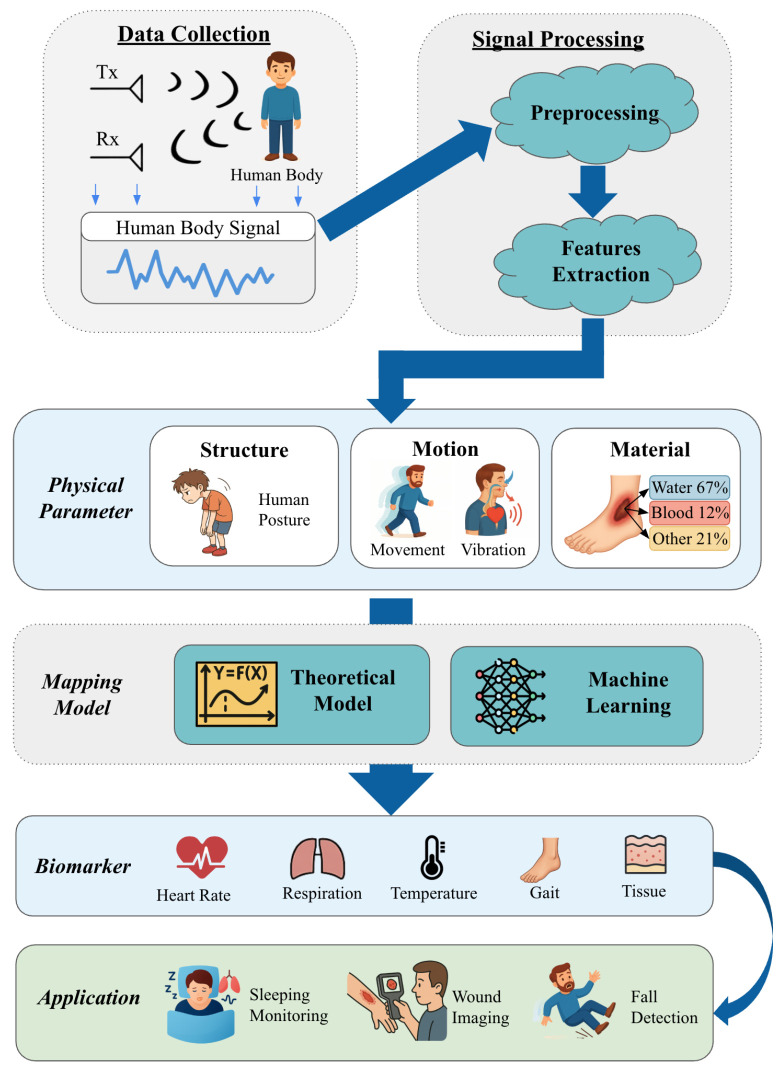
The framework of using mmWave technology for medical applications.

mmWave technology provides high-resolution, non-invasive methods for monitoring and diagnosing physiological conditions. As shown in Figure 2, a typical mmWave-based medical system includes system hardware, signal preprocessing, physical parameter extraction, parameter–biomarker mapping, and validation processes.

### 3.1. System Hardware Mechanism

mmWave systems leverage various hardware configurations to optimize sensing performance. Common modulation techniques include Continuous Wave (CW) and Frequency-Modulated Continuous Wave (FMCW), with FMCW enabling simultaneous range and velocity estimation. Antenna strategies such as Multiple-Input Multiple-Output (MIMO) and Synthetic Aperture Radar (SAR) enhance spatial resolution and imaging capability. Transceiver configurations can involve co-located or separated transmit and receive antennas, providing flexibility for diverse sensing scenarios.

### 3.2. Preprocessing

Signal preprocessing enhances the quality of mmWave data before analysis. Typical techniques include noise reduction (e.g., matched filtering), signal enhancement (e.g., low-noise amplification), and data compression to manage the large data volumes generated. These steps collectively improve the signal-to-noise ratio and facilitate efficient downstream processing [21,22].

### 3.3. Physical Parameter Extraction

mmWave sensing enables extraction of key physical parameters such as structural attributes (shape, size, texture), motion parameters (velocity, displacement), and material properties (dielectric constant, impedance). These parameters form the basis for mapping to medical relevant biomarkers. Details of parameter extraction methods are described in Section 4.

### 3.4. Parameter–Biomarker Connection

Mapping physical parameters to medical biomarkers can be achieved via theoretical models grounded in electromagnetic principles or data-driven machine learning approaches. This connection enables the estimation of health-related indicators from non-invasive mmWave measurements. Details of connection methods are described in Section 5.

### 3.5. Medical Biomarker Applications

Extracted biomarkers support a wide range of medical applications, including non-contact monitoring of physiological signals and assessment of tissue conditions. Detailed applications are discussed in Section 6.

### 3.6. Validation and Evaluation

Validation of mmWave-based medical systems involves phantom experiments [23], ex vivo testing [24], and in vivo studies [5], ensuring accuracy, reliability, and clinical relevance. These stages progressively bridge laboratory validation with real-world healthcare deployment.

## 4. Physical Parameters and Processing Strategies

In the context of mmWave technology, physical parameters refer to the measurable characteristics that influence how mmWave signals interact with materials and biological tissues. These parameters are crucial for designing and optimizing sensing systems and for enabling non-invasive medical applications. They can be broadly categorized into structural parameters, motion parameters, and material properties.

### 4.1. Structural Parameters

Structural parameters describe the geometric and surface characteristics of objects and tissues, including shape, size, roughness, and porosity. These parameters affect how mmWave signals are reflected and scattered, providing valuable information for tissue characterization and imaging. Techniques such as Synthetic Aperture Radar (SAR) and active mmWave imaging have been employed to extract these features from various materials [24,25].

### 4.2. Motion Parameters

Motion parameters characterize the dynamic behavior of biological tissues and body parts, encompassing both macro- and micro-scale movements. At the macro level, Doppler-based mmWave sensing enables the estimation of velocity, facilitating applications such as monitoring gross body movements and blood flow dynamics [26,27]. At the micro level, mmWave sensors are highly sensitive to minute displacements, enabling the analysis of physiological micro-vibrations. This capability supports the non-contact monitoring of vital signs, such as respiration and heart rate, through the detection of subtle chest wall movements associated with breathing cycles and cardiac activity [28,29].

### 4.3. Material Properties

Material properties, such as dielectric permittivity and absorption characteristics, directly influence mmWave signal propagation through biological tissues. These parameters can be used to differentiate tissue types and assess pathological changes. Recent studies have demonstrated the utility of mmWave-based measurements in evaluating tissue dielectric responses and absorption rates, providing insights into tissue health and composition [30,31].

## 5. Parameter–Biomarker Models

Parameter–biomarker models are essential tools in the field of medical diagnostics, research, and healthcare applications. These models aim to establish relationships between extracted mmWave physical parameters and medical biomarkers, which are indicative of specific physiological or pathological conditions. By quantifying these relationships, parameter–biomarker models enable the identification, tracking, and prediction of health conditions or disease progression. Generally, these models can be classified into two categories: Theoretical Models and Machine Learning Models. To better distinguish when and why a theoretical model or machine learning approach is preferable, Table 1 provides a structured comparison between these two categories. The details of these models are described in the following chapters.

### 5.1. Theoretical Model

A theoretical model connecting mmWave physical parameters to medical biomarkers involves understanding how mmWave signals interact with biological tissues and how these interactions can be quantitatively linked to specific physiological or pathological indicators. For example, Gabriel et al. [32] provide foundational data on tissue permittivity and conductivity across frequencies (including mmWave bands), which is essential for modeling the correlation between tissue material properties (e.g., absorption and reflectivity) and tissue biomarkers, such as tissue moisture and composition, which serve as indicators of tissue condition. Alekseev et al. [33] model mmWave absorption in blood vessels and correlate it with blood flow biomarkers (e.g., glucose, hemoglobin). Zhadobov et al. [34] review absorption mechanisms and thermal effects, linking power density to biomarker-related physiological changes. In mmWave sensing, the theoretical model is typically a valuable tool for bridging the gap between physical parameters and clinical biomarkers by enhancing the understanding of electromagnetic physics, thereby ensuring the system’s reliability and stability in medical applications.

### 5.2. Machine Learning

In addition to theoretical models, machine learning (ML) has emerged as a powerful tool for establishing connections between mmWave signal physical parameters and medical biomarkers. Unlike theoretical models, which are often based on predefined physical principles, machine learning methods can learn complex, non-linear relationships directly from data. For example, Iyer et al. [35] leverage mmWave sensor technology to monitor vital signs and detect arrhythmias in real time, employing machine learning techniques for precise analysis. Additionally, Bauder et al. [36] propose the use of end-to-end deep learning models to separate individual mmWave signals and directly map them to respiration patterns, enabling continuous respiration monitoring for patients with respiratory diseases, sleep apnea, or cardiovascular conditions.

## 6. Biomarkers and Their Medical Applications

**Figure 3:**
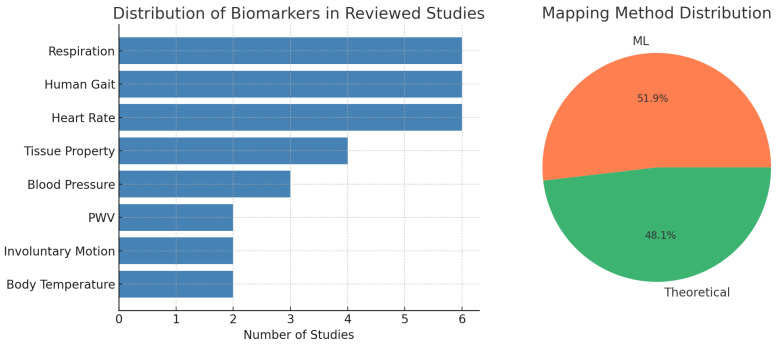
Summary statistics of 27 representative mmWave medical studies. (**Left**) Frequency of targeted biomedical biomarkers. (**Right**) Proportion of studies using theoretical vs. machine learning (ML)-based mapping models.

In mmWave-based medical systems, biomarkers are key physiological indicators extracted from the body through signal interactions. These include features such as respiration patterns, heart rate, blood pressure, and tissue properties. By linking physical features—like motion, material property, or structure—to these biomarkers, mmWave sensing enables various medical applications such as vital signs monitoring, cardiovascular assessment, gait analysis, and wound evaluation. Figure 3 summarizes the frequency of key biomarkers and the proportion of mapping methods adopted in 27 representative studies reviewed in this work. The most frequently targeted biomarkers are heart rate, respiration, and human gait. In terms of modeling strategy, machine learning and theoretical models are adopted in nearly equal proportions. This section introduces the commonly used biomarkers in mmWave sensing and their related clinical use cases, as summarized in Table 2.

### 6.1. Respiration Pattern

A significant body of work has explored mmWave sensing for extracting respiration patterns in a contactless manner. Most approaches leverage the chest wall’s micro-movements, which modulate the phase or amplitude of reflected mmWave signals. Early systems, such as Yang et al. [2] utilized 60 GHz signals and theoretical models to derive both respiration and heart rates from received signal strength variations. Subsequent works employed Doppler or FMCW radar to improve resolution and robustness. For instance, Iyer et al. [35] combined Fourier analysis and neural networks for arrhythmia detection using phase signals. More recent studies focus on classifying respiratory patterns using machine learning. Hao et al. [29] proposed mmWave-RM, which uses 77 GHz FMCW radar and image-based classifiers (e.g., SVM, CNN) to identify normal, quick, deep, and pathological (e.g., meningitic) breathing patterns. Beltrão et al. [56] further demonstrated reliable respiration monitoring for premature infants in neonatal intensive care units, addressing motion artifacts via harmonic-based signal decomposition. These systems showcase the capability of mmWave radar to accurately extract and classify respiration patterns across various clinical contexts, including sleep monitoring, apnea detection, and early screening of respiratory dysfunction.

### 6.2. Heart Rate

Recent advancements in mmWave radars have enabled accurate and contactless heart rate (HR) monitoring by detecting micro-movements of the chest wall induced by cardiac activity. Early systems typically relied on theoretical signal processing methods to extract phase or Doppler-based motion features. For instance, Chen et al. [40] proposed a DR-MUSIC algorithm to suppress respiratory harmonics and improve spectral resolution for precise HR estimation. Similarly, Hao et al. [42] designed an adaptive variational mode decomposition (A-VMD) method for isolating heartbeat signals, achieving stable performance even under motion interference. In parallel, deep learning-based models have emerged to enhance robustness and reduce latency, such as the mmArrhythmia system proposed by Zhao et al. [41], leverage encoder–decoder architectures and ensemble classifiers to estimate heart rate and classify arrhythmias, including atrial fibrillation and premature contractions. Together, these works demonstrate the potential of mmWave radar as a powerful tool for continuous, unobtrusive cardiac monitoring across both general health tracking and disease screening contexts.

### 6.3. Body Temperature

Recent studies have demonstrated that mmWave sensing can be effectively used for non-contact body temperature estimation by capturing thermal responses linked to dielectric or emission properties of materials. Chen et al. [13] introduced ThermoWave, a passive wireless sensing system that utilizes material property features—specifically the temperature-dependent thermal scattering of cholesteryl materials. This system maps scattering features to temperature using both a theoretical model (ThermoDot) and machine learning (GAN-based ThermoNet) for dot-wise estimation and thermal imaging, respectively. Separately, He et al. [55] developed a compact analog correlator-based mmWave radiometer that directly measures thermal emission power in the Ka band (32–36 GHz), using a theoretical correlation model to linearly infer body temperature from radiometric signals. These systems show strong potential for fever screening, skin temperature imaging, and contactless health monitoring, offering advantages over infrared sensors in terms of occlusion tolerance and environmental robustness.

### 6.4. Pulse Wave Velocity

Pulse Wave Velocity (PWV) is a critical biomarker reflecting arterial stiffness and is strongly correlated with blood pressure. Recent mmWave-based approaches enable non-contact estimation of PWV by capturing the transit delay of pulse waves across multiple body locations. Singh et al. [37] used a pair of FMCW radars placed at the chest and wrist to measure pulse transit time (PTT), which was converted to PWV for estimating systolic and diastolic blood pressure via polynomial regression and neural networks. Their method achieved root mean square errors below 3.7 mmHg and also explored waveform morphology (AUC) as a complementary blood pressure predictor. In a related study, Geng et al. [38] developed a stable mmWave pulse measurement system using a large MIMO radar array combined with range–angle focusing and phase unwrapping. By tracking arterial pulse waves at the neck, chest, and abdomen, the system demonstrated high temporal consistency and spatial resolution in waveform morphology, providing a reliable foundation for long-term cardiovascular monitoring based on PWV and pulse shape stability. Together, these studies highlight the feasibility of mmWave radar for continuous, contactless vascular assessment using PWV as a physiological bridge to blood pressure estimation.

### 6.5. Blood Pressure

Recent studies have demonstrated the feasibility of using mmWave radar for contactless blood pressure (BP) monitoring by extracting fine-grained arterial pulse information. Liang et al. [10] design the airBP to estimate both systolic and diastolic BP directly from the pulse waveform using a customized deep neural network with transformer-based attention, achieving FDA-compliant accuracy at up to 26 cm distance. In parallel, Hu et al. [12] introduced WaveBP, the first system to continuously reconstruct arterial blood pressure waveforms (ABPW) non-invasively from chest reflections. It combines mmWave signal modeling with a hemodynamics-informed hybrid transformer (mmFormer), multi-view beamforming augmentation, and cross-modal supervision from ECG/PPG data. WaveBP achieves high correlation (0.903) with true ABPW and supports detailed cardiac health assessment beyond static BP values. These works establish mmWave as a promising modality for unobtrusive, long-term cardiovascular monitoring in both home and clinical settings.

### 6.6. Tissue Property

mmWave systems have shown strong potential in non-invasive medical imaging by leveraging the dielectric property differences between normal and pathological tissues. These systems primarily extract material property features, such as dielectric constant or emissivity, to reconstruct tissue structure or quantify tissue damage. For instance, Bevacqua et al. [52] and Di Meo et al. [23] proposed mmWave-based breast imaging systems that use inverse scattering models and delay-and-sum radar algorithms to map tissue dielectric profiles, enabling the detection and characterization of malignant tumors in early-stage breast cancer. In skin cancer diagnostics, Mirbeik et al. [5] developed a high-resolution mmWave imaging (HR-MMWI) platform to classify malignant and benign skin lesions based on dielectric contrast, achieving up to 97% sensitivity in real-time, in vivo tests. Beyond oncology, Owda et al. [53] utilized mmWave radiometry to assess burn wounds by measuring changes in emissivity through bandages, revealing mmWave’s ability to monitor tissue healing and injury depth without dressing removal. Collectively, these studies demonstrate that mmWave sensing, through precise tissue property mapping, enables a range of diagnostic applications from cancer detection to wound evaluation, offering non-contact and high-resolution alternatives to traditional methods.

### 6.7. Human Gait

The mmWave radar has emerged as a powerful tool for non-contact gait analysis, offering high-resolution tracking of body motion while preserving privacy. Most approaches extract motion-related features, such as micro-Doppler signatures, step timing, stride length, or joint trajectories, to quantify gait characteristics. For instance, Alanazi et al. [46] developed a low-cost mmWave system with CNN-based classification to distinguish between five gait types, including abnormal walking patterns, achieving up to 98.8% accuracy. Jiang et al. [44] proposed a multi-channel 3D CNN model that utilizes point cloud motion trajectories from mmWave array radar to classify gait types in real time. Zeng et al. [45] focused on step time variability measurement in real-life environments, a key marker for early detection of cognitive decline and fall risk. Hu et al. [49] designed a mmWave-based system that reconstructs sit-to-stand movements using inverse kinematics and radar skeleton tracking to support fall risk assessment. Meanwhile, Zhang et al. [48] introduced mP-Gait, a fine-grained system for Parkinson’s disease monitoring that maps radar features to UPDRS-III gait scores using machine learning. These works demonstrate that mmWave sensing enables robust and scalable gait monitoring across use cases including elderly fall prevention, rehabilitation, and neurodegenerative disease assessment, outperforming traditional wearables in usability and privacy.

### 6.8. Involuntary Motion

The mmWave radar has shown strong potential in detecting and quantifying involuntary motions, such as tremors caused by neurological disorders. Gillani et al. [50] proposed a system that uses 77 GHz FMCW radar to extract motion features—specifically, the micro-vibrations of distal limbs—and quantifies tremor frequency and amplitude through a customized signal processing chain. The system maps radar phase and displacement signals to clinically meaningful biomarkers using a mathematical vibration model, enabling the accurate differentiation of essential tremor and Parkinsonian tremor across action, posture, and rest conditions. Experimental results showed high correlation ($R^{2}>0.97$) with reference sensors, with low mean errors (−0.14 Hz in frequency and −0.03 cm in amplitude), demonstrating strong feasibility for remote, unobtrusive tremor assessment. Complementing this, Smulders et al. [51] investigated millimeter-wave reflectometry to analyze tissue dielectric variation caused by hydration differences, indirectly linked to muscle or skin rigidity changes associated with involuntary motion. Their study confirmed that material property features—specifically, reflectivity magnitude in the 40–60 GHz band—can differentiate skin types and support applications like wound assessment or early-stage skin anomaly detection, even through bandages. Together, these studies highlight the potential of mmWave technologies serving as contactless tools for neurological monitoring and early detection tools of motor impairments.

## 7. Potential Applications

Beyond their established role in medical sensing, mmWave also shows considerable promise as a therapeutic modality for interacting directly with biological tissues. These interactions are governed by both thermal and non-thermal mechanisms, each with distinct biomedical implications. Thermal effects arise from the rapid absorption of mmWave energy by tissues, leading to highly localized heating and potential surface ablation [57]. In contrast, non-thermal effects—though less well understood—are garnering increasing scientific interest. Emerging evidence indicates that low-intensity mmWave exposure can influence cellular behavior without significant temperature elevation, affecting ion channel activity, membrane permeability, and even gene expression [34].

Building upon these underlying mechanisms, several therapeutic applications of mmWave radiation are under active investigation. For example, microwave ablation is a well-established clinical technique typically using sub-3 GHz frequencies to achieve deep tissue heating and tumor destruction [58]. However, the direct use of mmWave frequencies (30–300 GHz) for ablation remains largely experimental. Owing to their shallow tissue penetration, mmWaves are particularly well-suited for extremely localized and superficial ablation, making them promising for treating skin lesions, including potential applications in skin cancer [5,57]. Pilot studies have demonstrated cytotoxic effects on melanoma cells using 58–60 GHz exposures, although large-scale clinical validation is still pending. In addition to ablation, the localized thermal effects of mmWave energy have been explored for enhancing transdermal drug delivery, facilitating controlled diffusion across the skin barrier [59]. Meanwhile, non-thermal mechanisms have been implicated in the observed antimicrobial properties of mmWave exposure, with specific frequencies shown to inactivate bacteria and modulate antibiotic resistance profiles—offering a rapid, contact-free alternative to conventional surface sterilization methods [60].

Beyond these experimental applications, several mmWave-based therapeutic devices have also been commercialized. For instance, GradyVet MilliWave (Grady Medical, Murrieta, CA, USA) system is marketed for pain relief and inflammation reduction in soft tissue conditions through low-power mmWave exposure aimed at modulating inflammatory pathways [61]. In addition, wearable mmWave-emitting devices for neuromodulation in fibromyalgia patients have shown promising clinical results, including statistically significant improvements in patient-reported outcomes from multicenter randomized trials [62]. These developments highlight growing interest in applying mmWave technologies for therapeutic purposes such as pain management, inflammation reduction, immune system modulation, and wound healing.

## 8. Challenges and Future Directions

Despite rapid progress in mmWave-based medical sensing, several critical challenges remain open and present opportunities for future exploration:

### 8.1. Multimodal Fusion and Alignment

mmWave sensing can be combined with other modalities (e.g., visible, infrared, or depth sensors) to leverage complementary information, but this requires careful data alignment. For example, fusing mmWave radar with camera or thermal imagery can improve robustness and accuracy—as demonstrated by sensor-fusion systems in related domains—yet, aligning their data streams poses significant challenges. Accurate spatial calibration is needed to project radar-derived spatial information (e.g., range-angle points or point clouds) onto the image coordinate system. Temporal synchronization must also account for differing frame rates and sensor latencies. Moreover, heterogeneous spatial resolutions and non-overlapping fields of view further complicate fusion. Misalignment or clock drift can degrade performance, particularly in dynamic environments such as patient movement. Open problems include developing automatic calibration techniques—ideally self-calibrating or targetless—and learning-based alignment strategies that can generalize to new settings without manual intervention. Another key direction is ensuring robust fusion in the presence of degraded or missing data from one or more channels (e.g., camera occlusion or radar interference).

### 8.2. Model Generalization Across Subjects and Settings

Generalization to new patients and environments is a fundamental challenge. Models trained on one group of individuals or a specific room layout often suffer from “domain shift” when applied to others [63]. Variability in human anatomy (e.g., body size, posture), movement patterns, and clothing can alter the radar signatures of physiological signals or activities. Environmental factors such as multipath reflections and clutter further diversify data distributions. The medical machine learning literature highlights that without countermeasures, performance can degrade significantly under such distribution changes. Recent work has begun to address this via data-efficient learning; for instance, few-shot or meta-learning techniques enable a model to adapt quickly to a new user with only a few labeled examples. However, true robustness requires broader solutions such as domain-invariant feature learning or unsupervised domain adaptation, which remain largely unexplored for mmWave health sensing. Collecting larger, diverse datasets of patient radar data under varied conditions is critical. Future research should focus on developing models that explicitly account for inter-subject differences and environment variability, possibly by integrating synthetic augmentation or physics-based modeling with data-driven methods.

### 8.3. Interpretability and Explainability

As in many areas of medical AI, the “black-box” nature of deep learning models in radar-based sensing raises important concerns. Clinicians and regulators require transparency to ensure that medical decisions are understandable and justifiable. This is reinforced by policies such as the General Data Protection Regulation (GDPR), which mandates explainability for automated decisions that impact individuals [64]. mmWave radar data—such as Doppler spectrograms or range-angle maps—is not inherently interpretable by humans, which adds complexity. There is thus a need for explainable AI (XAI) techniques designed for radar inputs. Methods like attention mapping or feature attribution can highlight which signal components most influence a decision. Alternatively, using simpler models built on handcrafted features may improve interpretability, though often at a cost to accuracy. A key challenge is to balance performance with transparency, and to ensure that explanations remain faithful to the underlying physical principles of mmWave sensing (e.g., wave reflection, Doppler shift, and attenuation patterns).

### 8.4. On-Device and Real-Time Processing

Medical sensing often requires continuous, low-latency operation on embedded platforms (e.g., bedside monitors or wearables). While modern mmWave radar chips are extremely power-efficient, the computational load of signal processing and inference can be heavy. Real-time vital sign extraction and activity recognition typically involve multichannel FFTs, clustering, and neural-network inference, which may demand GPUs or FPGAs. Future directions include designing lightweight models (e.g., quantized or pruned networks) and specialized inference accelerators for radar data. Algorithmic optimizations—such as streaming signal processing, partial update schemes, or spiking neural networks—could further reduce latency. Ultimately, closing the gap between low-power radar hardware and the computing-intensive analytics remains a key challenge for practical medical devices.

### 8.5. Privacy and Federated Learning

Protecting patient privacy is essential in any medical sensing system. One advantage of the mmWave radar over traditional cameras is its ability to capture motion and physiological patterns without recording identifiable images. This makes mmWave especially suitable for use in environments like hospital rooms or homes, where preserving dignity and privacy is critical. Unlike video, radar signals reveal movement, posture, and breathing without showing the patient’s face or surroundings, which greatly reduces the risk of visual identity exposure. However, even without images, radar data can still carry sensitive health information. If collected and stored improperly, these signals could be used to infer medical conditions or personal habits. This creates challenges for data sharing and model training, especially when patient data is transmitted to central servers. Federated learning (FL) has emerged as a promising solution to this problem [65]. It allows devices or hospitals to collaboratively train models without exchanging raw data, reducing privacy risks. Still, care must be taken to ensure that the model itself does not leak sensitive information through its updates. Future work should continue to improve secure model training techniques, promote on-device inference, and explore methods such as encryption or differential privacy to ensure mmWave health systems remain safe and trustworthy.

### 8.6. Comparison with Vision-Based Modalities

Compared to conventional cameras, mmWave sensing presents distinct trade-offs for medical monitoring. Vision-based approaches such as RGB and thermal imaging offer high spatial resolution and rich contextual cues (e.g., facial expressions or skin appearance), making them useful for tasks like gesture recognition or skin condition analysis. However, these systems are highly sensitive to lighting conditions and introduce significant privacy concerns, particularly in long-term monitoring scenarios [64]. In contrast, mmWave radar operates reliably in the dark, through clothing, or in cluttered environments, and inherently preserves anonymity since it captures only range and motion data. Islam et al. [66] report that radar is often preferred over cameras in vital-sign monitoring tasks due to its non-invasive and privacy-preserving nature. The key limitation of mmWave sensing lies in its lower spatial resolution—the radar returns encode distance and motion but lacks fine-grained shape or texture details. While vision excels at capturing detailed appearance features, mmWave is more robust under occlusion and in dynamic lighting. As summarized in Table 3, each modality exhibits strengths and weaknesses across penetration capability, lighting robustness, spatial resolution, privacy, and hardware cost. Rather than viewing these as competing technologies, future systems should explore how to leverage them jointly—for example, combining mmWave’s robustness with visual detail from RGB or infrared sensors. Benchmarking studies that compare accuracy, real-time robustness, and patient experience across these modalities will be essential to guide their optimal use in healthcare.

### 8.7. Commercialization Outlook and Clinical Translation

Despite rapid progress in academic research, most mmWave-based medical technologies remain at the proof-of-concept or laboratory prototype stage, with limited industrial adoption to date. Key challenges include hardware integration, safety validation, regulatory approval, and cost-effectiveness compared to established clinical modalities such as ultrasound and infrared thermography.

Some sensing applications—such as body temperature estimation, blood pressure monitoring, and wound healing assessment—remain in the early stages of development and translation toward clinical use. While their clinical viability has yet to be fully established, several academic studies have demonstrated promising results in controlled experiments. For example, mmWave-based skin temperature estimation has been explored using surface reflectivity models, blood pressure prediction has leveraged chest wall motion and pulse wave velocity features, and wound healing progress has been assessed via changes in water content and tissue reflectivity. We view these directions as technically feasible and scientifically promising, though requiring further interdisciplinary effort and clinical validation to reach practical deployment.

## 9. Conclusions

In summary, this survey presents a comprehensive and structured view of mmWave-based medical sensing systems, spanning from signal acquisition and physical feature extraction to biomarker modeling and clinical deployment. By distinguishing low-level physical parameters (e.g., motion, material property, structure) from high-level biomedical biomarkers, and analyzing their mapping via both theoretical and ML-based approaches, we bridge the gap between wireless signal modeling and medical interpretation.

We envision this work serving as a foundational reference for future research in the following: (i) developing interpretable and adaptive modeling strategies; (ii) validating mmWave systems in clinically relevant and real-world environments; (iii) integrating mmWave with multimodal sensing technologies (e.g., thermal, optical, biosignals) for comprehensive health assessment.

The framework and taxonomy proposed in this work provide a reusable blueprint for researchers designing next-generation non-contact, real-time medical sensing solutions.

## Figures and Tables

**Table 1:** Comparison between Theoretical Model and Machine Learning in mmWave medical systems.

Aspect	Theoretical Model	Machine Learning (ML)
Modeling Principle	Physics-driven (e.g., wave propagation, reflectance equations)	Data-driven, learns feature–biomarker mappings
Interpretability	High—explicit and clinically traceable	Low to medium—requires explainability tools
Data Requirement	Low—relies on priors and assumptions	High—needs large labeled datasets
Adaptability	Limited—rigid to noise and variation	Flexible—handles subject and environmental variability
Accuracy	Stable in idealized settings, sensitive to real-world complexity	Higher potential in diverse conditions if well trained
Computational Demand	Low—suitable for embedded systems	Moderate to high—may require edge/cloud support
Medical Acceptance	High—transparent and regulator-friendly	Medium—needs explainability and validation
Use Case Preference	Simple vital signs under controlled setups	Complex tasks like gait, wound, or disease classification

**Table 2:** Summary of representative mmWave medical studies by feature type and biomarker.

Reference	Extracted mmWave Feature	Mapping Method	Target Biomarker	Medical Use Case
A. Motion Features				
Yang et al., 2017 [2]	Chest Micro-Variation	Theoretical	Respiration, Heart Rate	Sleep Monitoring
Iyer et al., 2022 [35]	Heart Micro-Vibration	ML	Respiration, Heart Rate	Arrhythmia Monitoring
Singh et al., 2023 [37]	Arterial Pulse Transit	ML	PWV, Blood Pressure	BP Monitoring
Hao et al., 2024 [29]	Chest Vibration	ML	Respiration	Vital Signs Monitoring
Geng et al., 2024 [38]	Artery Wall Vibration	Theoretical	PWV	Cardiovascular Monitoring
Wang et al., 2024 [39]	Chest Wall Movement	ML	Respiration	Sleep Apnea Detection
Chen et al., 2024 [40]	Chest Micro-Vibration	Theoretical	Heart Rate	Vital Signs Monitoring
Zhao et al., 2024 [41]	Chest Micro-Vibration	ML	Heart Rate	Arrhythmia Detection
Hao et al., 2025 [42]	Heart Vibration	Theoretical	Heart Rate	Vital Signs Monitoring
Mercuri et al., 2022 [43]	Chest Micro-Vibration	Theoretical	Respiration, Heart Rate	Vital Signs Monitoring
Jiang et al., 2020 [44]	Radar Point-Cloud + Micro-Doppler	ML	Human Gait	Gait Monitoring
Zeng et al., 2022 [45]	Step Timing (Micro-Doppler)	Theoretical	Human Gait	Gait Monitoring
Alanazi et al., 2022 [46]	Gait Micro-Movement	ML	Human Gait	Rehabilitation Monitoring
Feng et al., 2023 [47]	Limb Movement	ML	Human Gait	Elderly Fall Detection
Zhang et al., 2024 [48]	Stride/Gait Velocity	ML	Human Gait	Parkinson’s Assessment
Hu et al., 2024 [49]	Joint Kinematics	ML	Human Gait	Fall Risk Assessment
Gillani et al., 2023 [50]	Limb Tremor Vibration	Theoretical	Involuntary Motion	Parkinson’s Detection
Smulders et al., 2013 [51]	Skin Water Content	Theoretical	Involuntary Motion	Tremor Monitoring
B. Structure and Material Features				
Bevacqua et al., 2021 [52]	Dielectric + Geometry	Theoretical	Tissue Property	Breast Cancer Imaging
Di Meo et al., 2021 [23]	Dielectric + Geometry	Theoretical	Tissue Property	Breast Cancer Imaging
Mirbeik et al., 2022 [5]	Dielectric Constant	ML	Tissue Property	Skin Cancer Diagnosis
Owda et al., 2019 [53]	Emissivity	Theoretical	Tissue Property	Burn Wound Assessment
Bagheri et al., 2024 [54]	Dielectric Constant	Theoretical	Respiration	Vital Signs Monitoring
Chen et al., 2020 [13]	Thermal Scattering (Polymer Film)	ML	Body Temperature	Fever Screening
He et al., 2023 [55]	Thermal Emission (Body)	Theoretical	Body Temperature	Fever Screening
Liang et al., 2023 [10]	Blood Volume Change	ML	Blood Pressure	BP Monitoring
Hu et al., 2024 [12]	Chest Micro-Vibration	ML	Blood Pressure	BP Monitoring

Note: ML = Machine Learning; PWV = Pulse Wave Velocity.

**Table 3:** Comparison of mmWave, infrared, and optical imaging in medical sensing.

Aspect	mmWave Sensing	Infrared Imaging	Optical Imaging
Penetration Capability	High—penetrates clothing, gauze	None—blocked by barriers	None—surface only
Lighting Sensitivity	Not affected by ambient lighting	Low—robust to illumination	High—requires consistent lighting
Privacy	High—non-visual, anonymous	Medium—thermal silhouettes possible	Low—captures identifiable appearance
Spatial Resolution	3–10 mm	1–4 mm	<0.1 mm
Hardware Cost	$100–500	$200–800	$10–50

Note: Resolution and cost are approximate, based on typical hardware used in non-contact sensing at 0.3–1 m distance.
